# Investigating the Impact of Maternal Obesity on Disease Severity in a Mouse Model of Preeclampsia

**DOI:** 10.3390/nu17091586

**Published:** 2025-05-05

**Authors:** Natalie K. Binder, Natasha de Alwis, Bianca R. Fato, Sally Beard, Yeukai T. M. Mangwiro, Elif Kadife, Fiona Brownfoot, Natalie J. Hannan

**Affiliations:** 1 Therapeutics Discovery & Vascular Function in Pregnancy Group, University of Melbourne, Mercy Hospital for Women, Heidelberg 3084, Australia; nkbinder@unimelb.edu.au (N.K.B.); natasha.dealwis@unimelb.edu.au (N.d.A.); bianca.fato@unimelb.edu.au (B.R.F.); beard.s@wehi.edu.au (S.B.); yeukai.mangwiro@unimelb.edu.au (Y.T.M.M.); 2 The Walter and Eliza Hall Institute of Medical Research, Parkville 3050, Australia; 3 Obstetrics Diagnostics and Therapeutics Group, University of Melbourne, Mercy Hospital for Women, Heidelberg 3084, Australia; elif.kadife@unimelb.edu.au (E.K.); fiona.brownfoot@unimelb.edu.au (F.B.)

**Keywords:** preeclampsia, obesity, pregnancy, endothelial dysfunction, mouse model

## Abstract

Background: Preeclampsia is a leading cause of maternal and fetal morbidity and mortality, with obesity recognised as a significant risk factor. However, the direct contribution of obesity to the pathophysiology underpinning preeclampsia remains unclear. Objectives: This study aimed to develop and characterise a diet-induced obese mouse model with superimposed preeclampsia to better understand the impact of obesity on disease pathogenesis. Methods: Female mice were fed either standard rodent chow or a high-fat diet from weaning. At 8 weeks of age, mice were mated. Pregnant mice were treated with L-N^G^-Nitro arginine methyl ester (L-NAME; to block nitric oxide production) from gestational day (D)7.5 to D17.5 to induce a preeclampsia-like phenotype. Blood pressure was measured on D14.5 and D17.5, followed by the collection of maternal and fetal tissues for histological, biochemical, and molecular analyses. Results: Obese dams exhibited significantly increased body, fat pad, and liver weights compared to lean controls. While L-NAME induced hypertension in the control mice, contrary to expectations, the L-NAME-induced hypertension was partially attenuated in obese dams, with significantly lower systolic and diastolic blood pressures at D14.5 and reduced systolic pressure at D17.5. Fetal weights were comparable between groups, however, placentas were significantly heavier with obesity. Endothelial function, inflammatory markers, and renal gene expression patterns suggested distinct physiological adaptations in obese preeclamptic-like mice. Conclusions: These findings challenge the prevailing assumption that obesity drives hypertension, endothelial dysfunction, and inflammatory markers. The differential vascular and physiological responses observed in the obese dams highlight the complexity of obesity–preeclampsia interactions and underscore the need for refined preclinical models to disentangle mechanistic contributions. This work has implications for personalised management strategies and targeted therapeutic interventions in obese pregnancies at risk of preeclampsia.

## 1. Introduction

Preeclampsia is a leading cause of maternal and fetal morbidity and mortality, affecting 2–8% of all pregnancies [1,2,3]. Although the exact aetiology of preeclampsia remains unclear, it is widely recognised as a disorder of placental dysfunction and systemic endothelial impairment, likely driven by ischemia–reperfusion injury of the placenta [4,5], creating a hypoxic placental environment and triggering the release of anti-angiogenic and pro-inflammatory factors into the maternal circulation [6,7]. Obesity has long been identified as a significant risk factor for preeclampsia, with a dose-dependent relationship between increasing body mass index (BMI) and disease incidence [8,9,10,11]. In high-income countries, up to 30% of pregnant individuals are classified as obese (BMI ≥ 30 kg/m^2^), with prevalence varying by socioeconomic status and ethnicity [12,13]. Given the global rise in obesity, particularly among reproductive-aged individuals [12,14,15,16,17], the contribution of maternal adiposity to preeclampsia is of increasing concern.

The link between obesity and preeclampsia is thought to be mediated by chronic inflammation [18,19], oxidative stress [20,21], and dysregulated angiogenic signalling [22], all of which can contribute to endothelial dysfunction and abnormal placental development [23,24,25,26,27]. Although the processes associated with obesity are well-characterised, the extent to which obesity directly contributes to preeclampsia pathophysiology remains debated; not all obese individuals develop preeclampsia, and not all individuals who develop preeclampsia are obese. Recent evidence suggests that having a higher BMI in a pregnancy complicated by preeclampsia is linked to increased disease severity, reflected by higher rates of placental malperfusion [28].

Preclinical models are essential to better understand the physiological processes of obesity-associated preeclampsia. Murine models have been extensively utilised to mimic key features of preeclampsia, including hypertension, fetal growth restriction, proteinuria, and placental abnormalities [29,30,31,32,33]. However, the one existing model of obesity with superimposed preeclampsia is based on genetically obese mice [34,35,36], despite evidence that genetic and diet-induced obesity elicits distinct metabolic and cardiovascular responses in pregnancy [37]. Here, we demonstrate the development of a Western-style high-fat diet-induced obese mouse model with superimposed preeclampsia. Unexpectedly, our findings challenge the prevailing assumption that obesity universally worsens preeclampsia physiology and symptoms, as the obese group demonstrated fewer adverse outcomes than their lean counterparts. This highlights the complexity of obesity’s interaction with preeclampsia and underscores the need for refined models to dissect its mechanistic contributions.

By evaluating the physiological and molecular changes in this model, we aim to provide new insights into the nuanced relationship between maternal obesity and preeclampsia, with implications for both clinical management and future therapeutic strategies.

## 2. Methods

### 2.1. Animal Studies

All animal procedures adhered to the ethical standards outlined by the National Health and Medical Research Council and received approval from our institutional Animal Ethics Committee (A2018/05596; approved 26 February 2019, Austin Health). Three-week-old female CBA × C57BL/6 (F1) mice (n = 20, Florey Institute) were maintained at 18–22 °C and 50% relative humidity under a 12 h light/dark cycle. Access to food and water was unrestricted. At 4 weeks old, mice were allocated at random to standard rodent chow or a high-fat diet designed to emulate a Western-style fast food diet (40% energy from fat; SF05-31, Specialty Feeds, Glen Forrest, Australia).

At 8 weeks old, female mice were weighed to ensure substantial weight gain due to the high-fat diet and then mated overnight with male F1 mice. A copulatory plug confirmed mating the next morning, marking the beginning of gestational day (D)0.5.

The nitric oxide synthase inhibitor, L-N^G^-Nitro arginine methyl ester (L-NAME, Sigma-Aldrich, St. Louis, MO, USA), was administered daily (50 mg/kg/day; 100 µL subcutaneous) from D7.5 to D17.5 of gestation (similar to the second and third trimester in humans) to induce a preeclampsia-like phenotype in the pregnant mice [30,31].

### 2.2. Blood Pressure Measurements

Prior to pregnancy, mice were trained on the CODA tail-cuff system (Kent Scientific, Torrington, CT, USA) for non-invasive blood pressure measurements using a stepwise acclimation protocol that included tube restraint. Blood pressure measurements were taken on D14.5 and D17.5, 30 min after daily L-NAME injection. Mice were pre-warmed on a heated platform and acclimated to the device (tube restraint and tail cuff) for 15 min prior to measurements beginning. A total of 25 blood pressure measurement cycles were carried out per mouse per timepoint. The average of these cycles, excluding those with excessive movement artefacts, was used for statistical analysis.

### 2.3. Tissue Collection

Following final blood pressure measurements, dams were weighed and maternal blood collected via cardiac puncture in anaesthetised mice (5% isoflurane in oxygen), before being humanely killed by cervical dislocation. Coagulated blood samples were centrifuged at 5000× *g* for 15 min at room temperature, and the serum was collected and snap-frozen for storage at −80 °C.

Fetuses were weighed, and digital callipers were used to measure crown-to-rump length. Gonad morphology was used to determine fetal sex. Umbilical cords and fetal membranes were removed from each placenta before weighing. Dam kidneys were preserved in RNAlater for at least 48 h and snap-frozen for storage at −80 °C. Dam kidneys and livers were fixed overnight in 4% paraformaldehyde for histological analysis. Dam intestines were collected in PBS and maintained on ice for dissection of the mesenteric arteries used in vascular studies. Dam intraperitoneal adipose tissue was weighed.

### 2.4. Dam Kidney and Liver Histology

Fixed livers and kidneys were processed and embedded in paraffin blocks. Tissue sections, 4 µm thick, were deparaffinised in xylene, then rehydrated through descending concentrations of ethanol prior to staining with haematoxylin and eosin. A Nikon Eclipse Ci microscope and camera (Nikon, Minato City, Japan) were used to capture histological images at 400× total magnification (n = 3 sections/condition).

For kidney analysis, glomeruli size and cellularity, immune infiltration, tubule structures, and necrosis and apoptosis were evaluated. For liver analysis, steatosis features such as nuclear displacement, immune infiltration, necrosis, and fibrosis were assessed. High-magnification images of the regions of interest are shown in the figures, illustrating the overall impact of obesity with superimposed preeclampsia.

### 2.5. Vascular Reactivity Studies

Mesenteric arteries (second-order) were isolated and cleaned of connective and adipose tissue in Krebs physiological salt solution (NaCl 120 mM, KCl 5 mM, MgSO_4_ 1.2 mM, KH_2_PO_4_ 1.2 mM, NaHCO_3_ 25 mM, D-glucose 11.1 mM, CaCl_2_ 2.5 mM). Arteries were cut into 2 mm long segments and mounted on a Wire Myograph (620 M; Danish Myo Technology (DMT), Hinnerup, Denmark) using gold-plated tungsten wires (25 µm diameter, W005230; Goodfellow, Cambridge, UK). Mounted vessels were immersed in continuously carbogenated (95% O_2_, 5% CO_2_) Krebs salt solution and maintained at 37 °C. Arteries were normalised to 100 mmHg (13.3 kPa) pressure using LabChart v8.1.21 software (ADInstruments, Sydney, Australia) employing the DMT normalisation module with IC1/1C100 = 1. To assess smooth muscle viability, vessel segments were exposed to high potassium physiological salt solution (KPSS; NaCl 25 mM, KCl 100 mM, MgSO_4_ 1.2 mM, KH_2_PO_4_ 1.0 mM, NaHCO_3_ 25 mM, D-glucose 11.1 mM, CaCl_2_ 2.5 mM). Endothelial integrity was evaluated by pre-constricting arteries with phenylephrine (Sigma-Aldrich) to 50–70% of their maximum KPSS-induced tone, followed by relaxation with endothelial-dependent dilator, acetylcholine (Sigma-Aldrich). Vessels that did not achieve at least 80% relaxation were excluded. Dose–response curves for vasoconstriction and vasorelaxation were generated using cumulative concentrations of phenylephrine and acetylcholine (10^−9^ to 10^−4.5^ M).

### 2.6. Enzyme-Linked Immunosorbent Assay (ELISA)

Dam serum concentrations of soluble fms-like tyrosine kinase 1 (sFLT-1), endothelin-1 (ET-1), and C-reactive protein (CRP) were quantified with enzyme-linked immunosorbent assay (ELISA) kits (R&D Systems, Minneapolis, MN, USA), according to manufacturer instructions. Specifically, the Mouse sVEGFR1/Flt-1 DuoSet kit was used with samples diluted 1:100, while the Mouse Endothelin-1 Quantikine and Mouse CRP Quantikine kits were used with neat samples.

### 2.7. Quantitative Polymerase Chain Reaction (qPCR)

Total RNA was isolated from kidneys using the RNAeasy extraction kit (Qiagen, Hilden, Germany). RNA concentration and quality were determined using a Nanodrop 2000 spectrophotometer (ThermoFisher, Waltham, MA, USA). cDNA synthesis was carried out using the High-Capacity cDNA Reverse Transcription Kit (Applied Biosystems, Waltham, MA, USA) on the iCycler iQ5 (Bio-Rad, Hercules, CA, USA). Quantitative real-time PCR was performed using TaqMan reagents and primers (Life Technologies, Carlsbad, CA, USA). Target transcripts included fibronectin 1 (*Fn1*; Mm01256744_m1), hydroxysteroid 11-beta dehydrogenase 2 (*Hsd11b2*; Mm01251104_m1), NADPH oxidase 4 (*Nox4*; Mm00479246_m1), serum/glucocorticoid regulated kinase 1 (*Sgk1*; Mm00441380_m1), sodium channel epithelial 1 subunit alpha (*Scnn1α*; Mm00803386_m1), with reference gene ubiquitin C (*Ubc*; Mm01198158_m1).

### 2.8. Statistical Analysis

Data were first evaluated for normality using Gaussian distribution assessments. Depending on data distribution and variance, either unpaired t-tests (parametric) or Mann–Whitney U tests (non-parametric) were used to compare lean and obese groups. Linear mixed-effects models were applied to assess fetal and placental measurements, incorporating treatment group as a fixed effect and litter as a random effect. Significance of treatment-related differences was determined via nested ANOVA. For vascular reactivity studies, dose–response curves were generated using four-parameter non-linear regression (log[agonist] vs. response). Comparisons across concentration–response curves were analysed with mixed-effects models, and multiple comparisons were adjusted using the Šidák method. A *p*-value threshold of <0.05 was considered indicative of statistical significance. All analyses were conducted using GraphPad Prism version 8 (GraphPad, Boston, MA, USA).

## 3. Results

### 3.1. Body and Organ Weights Were Elevated in Obese Dams

**Figure 1:**
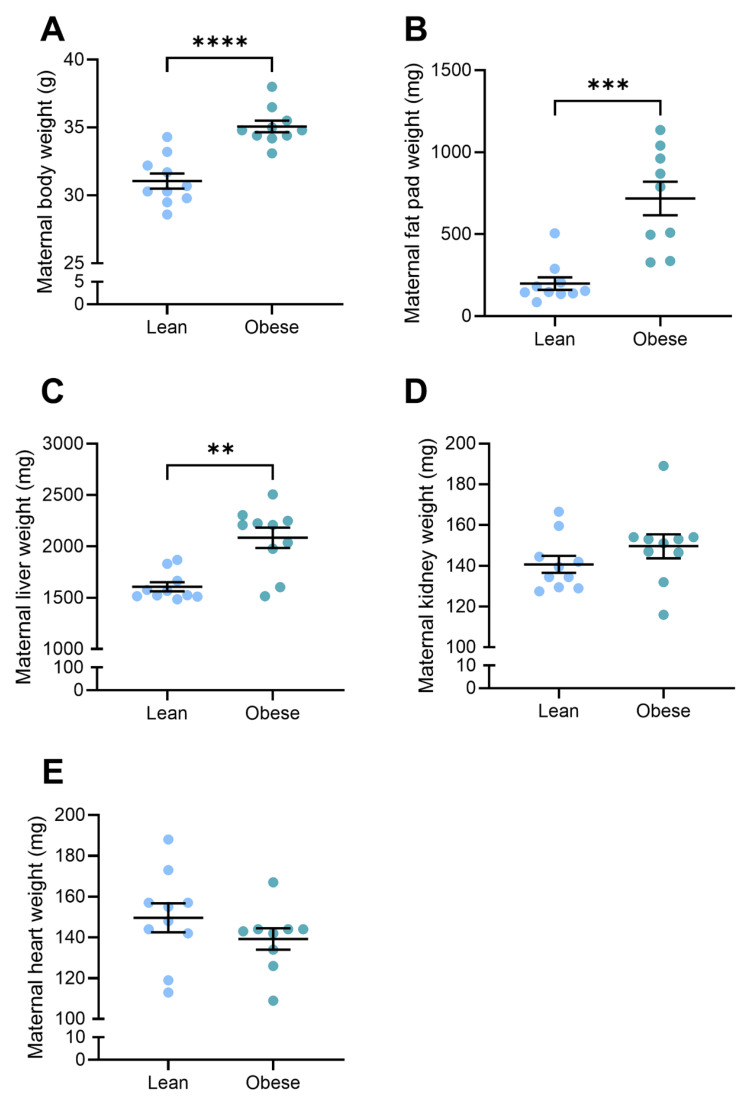
Maternal body and organ weight at D17.5 of pregnancy. (**A**) Maternal body, (**B**) fat pad, and (**C**) liver weights were higher in the mice on a high-fat diet (obese). (**D**) Maternal kidney and (**E**) heart weights were not different between the lean (standard diet) and obese mice. Mean ± SEM, n = 10/group. ** *p* < 0.01, *** *p* < 0.001, **** *p* < 0.0001.

Immediately prior to mating, mice receiving a high-fat diet for four weeks weighed significantly more than mice receiving standard chow diet (19.7 ± 0.32 g vs. 13.9 ± 0.45 g; *p* = 0.0001) and were designated as obese and lean, respectively (Supplementary Figure S1). At D17.5 of pregnancy, obese mice had significantly elevated body weight (35.1 ± 0.43 g vs. 31.1 ± 0.56 g; *p* < 0.0001), fat pad weight (718.6 ± 102.4 mg vs. 199.2 ± 38.1 mg; *p* = 0.0002), and liver weight (2083 ± 98.4 mg vs. 1607 ± 43.6 mg; *p* = 0.0015) compared to lean mice (Figure 1A–C). There were no differences in dam kidney or heart weights (Figure 1D,E) between the two groups.

### 3.2. Blood Pressure Was Reduced in Obese, L-NAME-Treated Dams

**Figure 2:**
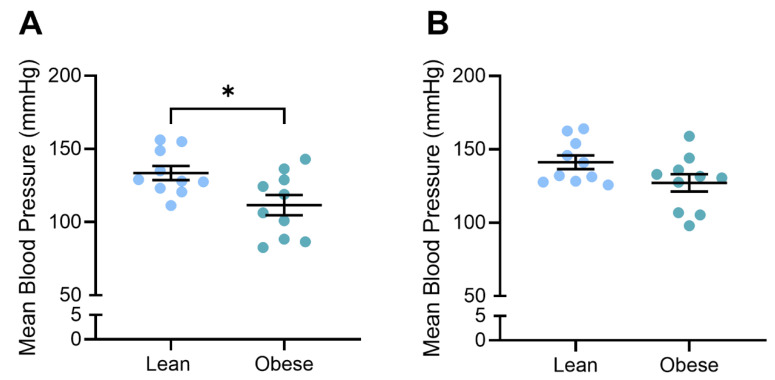
Mean arterial blood pressure of lean and obese mice at (**A**) D14.5 and (**B**) D17.5. Blood pressure was measured via tail cuff plethysmography. At D14.5, mean arterial pressure was lower in the obese dams compared to the lean mice (**A**). At D17.5, there was no difference between lean and obese dams (**B**). Mean ± SEM, n = 10/group. * *p* < 0.05.

We previously demonstrated that L-NAME administration to F1 mice significantly elevated blood pressure at both D14.5 and D17.5 gestation [31], modelling a key characteristic of preeclampsia. Here, we demonstrate that obese L-NAME-treated mice had lower blood pressure than lean L-NAME-treated mice: significantly lower systolic (*p* = 0.0247) and diastolic (*p* = 0.0171) at D14.5 and lower systolic blood pressure at D17.5 (*p* = 0.0434) (Supplementary Figure S2A–C). Diastolic blood pressure was not significantly different in the obese compared to lean mice at D17.5 (Supplementary Figure S2D). As a result, mean arterial pressure was significantly lower in the obese mice at D14.5 (*p* = 0.018; Figure 2A) but not different to the lean mice at D17.5 (Figure 2B).

### 3.3. Circulating Vasoconstrictor Levels Were Reduced in Obese Dams

**Figure 3:**
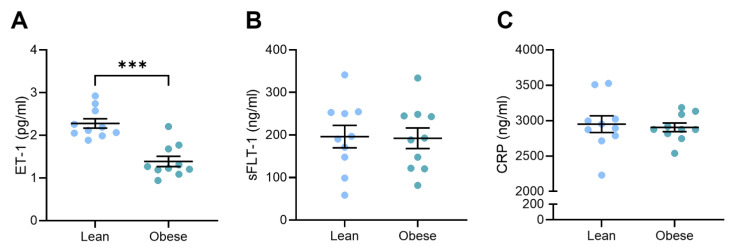
Levels of circulating factors associated with preeclampsia in lean and obese mice administered L-NAME. Endothelin-1 (ET-1), soluble fms-like tyrosine kinase (sFLT-1), and C-reactive protein (CRP) were measured via ELISA. Circulating ET-1 levels were significantly lower in the obese dams compared to the lean dams (**A**). There was no difference in sFLT-1 (**B**) or CRP (**C**) levels between the lean and obese groups. Mean ± SEM, n = 10/group. *** *p* < 0.001.

Our team has previously shown that L-NAME administration increases circulating levels of factors associated with preeclampsia pathogenesis, including vasoconstrictor ET-1, anti-angiogenic factor sFLT-1, and marker of inflammation, CRP [31]. Here, we found that circulating vasoconstrictor ET-1 levels were significantly lower in the obese dams compared to the lean dams (*p* = 0.0003; Figure 3A). There was no difference in circulating anti-angiogenic sFLT-1 (Figure 3B) or inflammatory CRP (Figure 3C) levels between the lean and obese dams.

### 3.4. Obese Mice Had Increased Vasorelaxation Compared to Lean Mice

**Figure 4:**
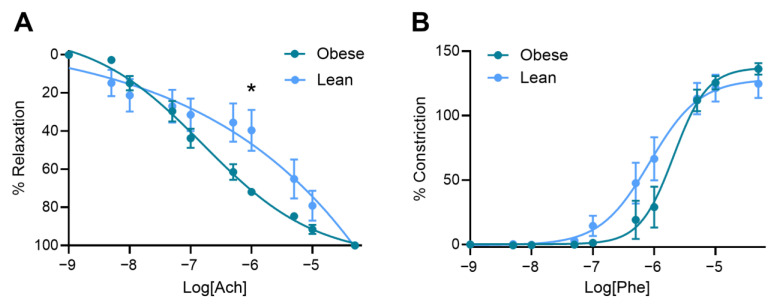
Vascular reactivity of mesenteric arteries collected from obese and lean mice treated with L-NAME. Vasorelaxation to acetylcholine (Ach) (**A**) and vasoconstriction to phenylephrine (Phe) (**B**) were measured via wire myography. Vasorelaxation was significantly increased for mesenteric arteries collected from obese dams at 10^−6^ M Ach compared to lean mice. There was no difference in mesenteric artery vasoconstriction to Phe between the groups. Curve is a non-linear regression log[agonist] vs. response (variable slope—four parameters). Mean ± SEM, n = 6–7/group. * *p* < 0.05. Maximum constriction, area under the curve, and logEC50 are presented in Supplementary Figure S3.

Mesenteric arteries collected from obese dams demonstrated enhanced vasorelaxation in response to acetylcholine, including significantly elevated vasorelaxation at 10^−6^ M acetylcholine (*p* = 0.0108; Figure 4A) and a lower LogEC50 (*p* = 0.0411; Supplementary Figure S3A), meaning the artery relaxed at a lower dose of acetylcholine). There was no significant difference in area under the curve (Supplementary Figure S3B) or maximum vasorelaxation (Rmax; Supplementary Figure S3C).

The mesenteric arteries collected from the obese and lean mice did not differ in vascular response to phenylephrine: no difference was observed at any dose of phenylephrine assessed (Figure 4B), nor LogEC50, area under the curve, or maximum constriction (Emax) (Supplementary Figure S3D–F).

### 3.5. Obesity with Superimposed Preeclampsia Alters Placental Size

**Figure 5:**
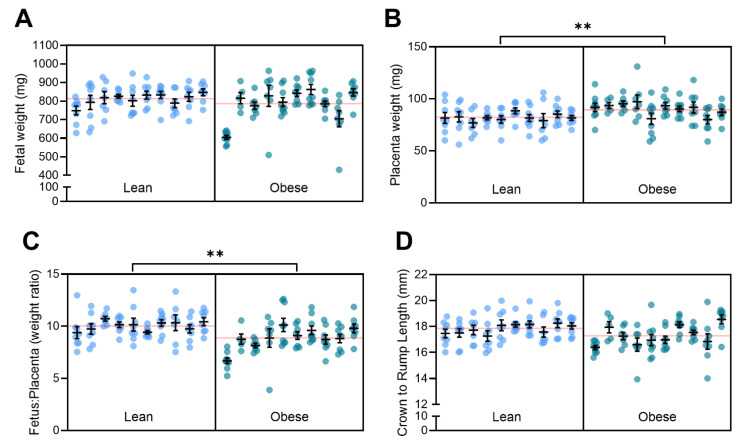
Effect of diet on fetal and placental size. Fetal weight (**A**) was not significantly different between the litters of obese and lean dams. Placental weight (**B**) was significantly elevated in the obese group, and the fetal-to-placental weight ratio (**C**) decreased compared to the lean group. Crown-to-rump length (**D**) measured via callipers was not altered between the litters of each group. Each sub-column presents pups from a single dam. Red line across each box presents the mean of the group. Mean ± SEM, n = 10 dams/group. ** *p* < 0.01.

Our team has previously shown that L-NAME administration decreases pup size and placental weight [31]. Here, we found that the D17.5 fetuses of obese dams did not have altered weight compared to the fetuses of lean dams (Figure 5A). However, the obese mice had significantly heavier placentas (*p* = 0.0014; Figure 5B) and a decreased fetal-to-placental weight ratio (*p* = 0.0034; Figure 5C). These results (fetal and placental weight) were consistent between male and female fetuses (Supplementary Figure S4A–F).

The crown-to-rump length of the D17.5 fetuses was lower in the obese group but not significantly different to the fetuses of the lean mice (Figure 5D). However, splitting the data by fetal sex demonstrated that though there was no difference in the crown-to-rump length in female fetuses (Supplementary Figure S4G), the male fetuses from the obese dams had significantly reduced crown-to-rump length compared to the lean group (*p* = 0.0242; Supplementary Figure S4H).

### 3.6. Obese Mice Demonstrated Altered Expression of Genes Associated with Kidney Function

**Figure 6:**
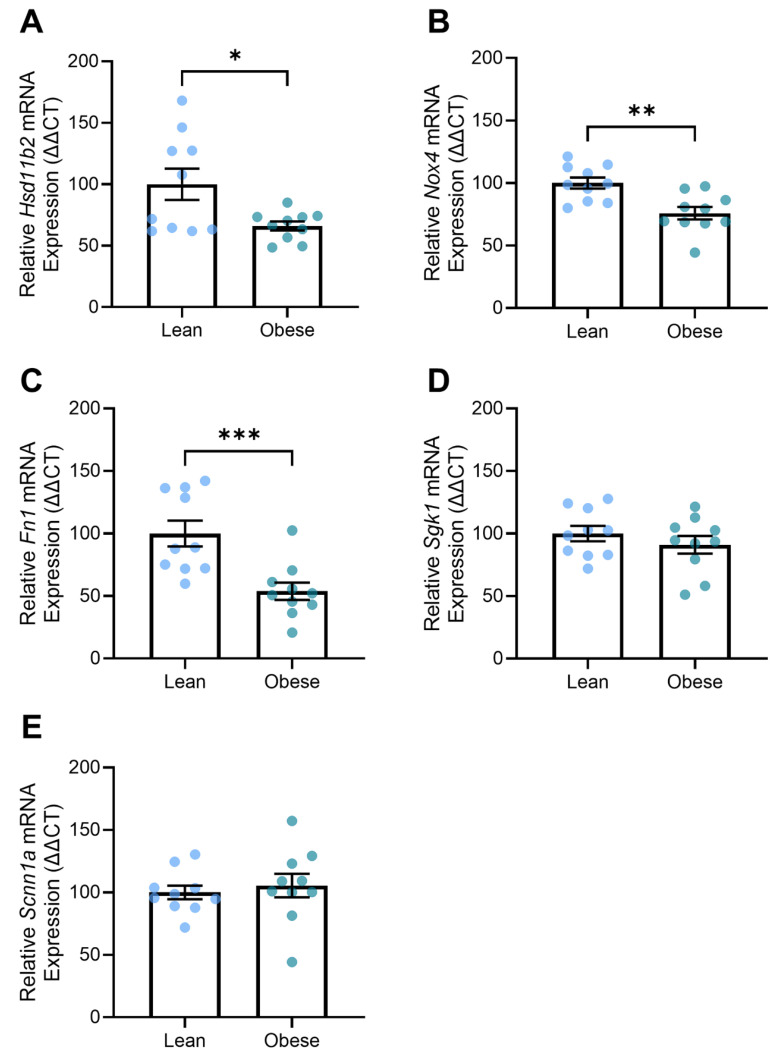
Effect of diet on expression of genes involved in kidney function in obese pregnant mice receiving L-NAME. Gene expression was measured via PCR. Expression of *Hsd11b2* (**A**), *Nox4* (**B**), and *Fn1* (**C**) were lower in kidneys from obese dams compared to those from lean mice. *Sgk1* (**D**) and *Scnn1a* (**E**) expression were not different between the obese and lean groups. Mean ± SEM, n = 10 dams/group. * *p* < 0.05, ** *p* < 0.01, *** *p* < 0.001.

One of the clinical features of preeclampsia can be proteinuria due to renal damage. Here, we found that the obese L-NAME-treated mice had decreased renal expression of genes related to kidney function, including *Hsd11b2* (*p* = 0.02; Figure 6A), *Nox4* (*p* = 0.0019; Figure 6B), and *Fn1* (*p* = 0.0007; Figure 6C). The expression of genes involved with fluid balance, electrolyte transport, and mineralocorticoid stimulation, *Sgk1* and *Scnn1a*, were not altered between the groups (Figure 6D,E).

### 3.7. Kidney and Liver Histology

**Figure 7:**
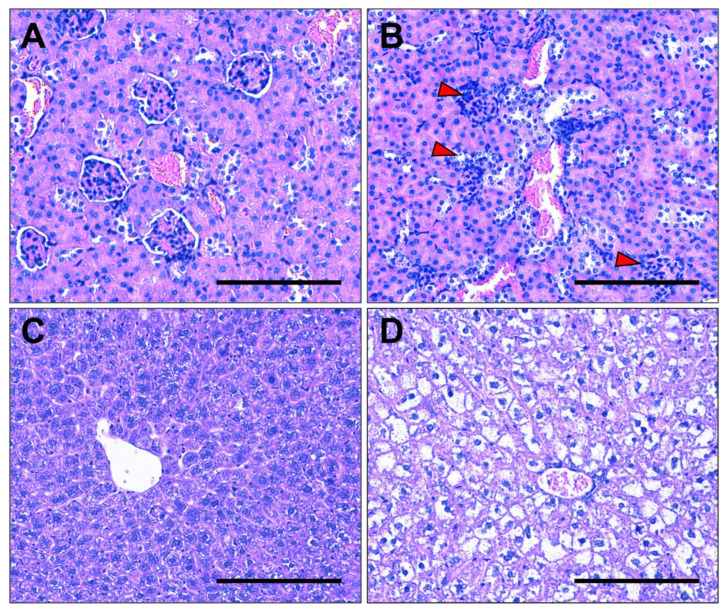
Pathological changes in the kidneys and livers of obese pregnant mice receiving L-NAME. Haematoxylin and eosin-stained kidney sections from lean (**A**) and obese (**B**) dams. The kidneys of obese dams demonstrated enlarged glomeruli, narrowing of the Bowman’s capsule, and immune infiltrations (red arrows). Haematoxylin and eosin-stained liver sections from lean (**C**) and obese (**D**) dams. Livers from obese dams had steatosis with nuclear displacement. Scale bar = 100 µm.

Obesity exacerbated pathological changes in the liver and kidneys of L-NAME mice (Figure 7A–D). In the kidneys, obesity led to glomerular swelling, increased cellularity, and narrowing of Bowman’s space (Figure 7A,B). There was also an increase in immune infiltration and irregular kidney tubules in the obese L-NAME-treated mice. In the obese livers, steatosis was observed, with lipid accumulation in hepatocytes that displaced or caused a complete loss of nuclei. Additionally, some regions showed potential fibrosis (Figure 7D).

## 4. Discussion

The interplay between obesity and preeclampsia presents a complex and multifaceted issue that remains inadequately understood. While obesity is strongly linked to an increased incidence of preeclampsia [8,9,10,11], its impact on disease severity remains less well-defined. Clarifying this relationship is crucial, as it could reveal whether simple lifestyle modifications might help mitigate the risk or severity of preeclampsia, offering a practical avenue for intervention. This study aimed to establish a mouse model of diet-induced obesity with superimposed preeclampsia to enhance our understanding of disease pathophysiology. While there is some evidence in the literature of increasing BMI being associated with increased preeclampsia severity [28], our findings provide important insights that challenge this assumption. Specifically, we observed that obesity, in the context of preeclampsia, appeared to improve several key parameters traditionally associated with the condition, including blood pressure, endothelial function, and placental weight.

Unexpectedly, a key finding of this study was significantly decreased gestational blood pressure with obesity compared to lean controls. This reduction in blood pressure contradicts the well-established positive correlation between blood pressure and BMI [38] or the up to 75% increased risk of developing hypertension in obese individuals [39]. Notably, while some studies suggest a dose-dependent relationship between obesity and preeclampsia incidence [8,9,10,11], there is a lack of consistent experimental data linking obesity directly to hypertension in the context of preeclampsia. Our findings suggest that, at least in this animal model, the metabolic and inflammatory milieu induced by obesity may have protective effects in superimposed preeclampsia. Further studies in additional models of preeclampsia are needed to confirm and extend these findings in the context of obesity.

In line with this, obesity appeared to reduce the impact of L-NAME on endothelial function, as evidenced by improved vasorelaxation in response to acetylcholine, a key mediator of endothelial-dependent relaxation [40]. This finding contrasts with the expected endothelial dysfunction typically associated with both obesity and preeclampsia, where inflammation, oxidative stress, and impaired angiogenesis are thought to disrupt normal vascular responses [18,19,20,22,23,24,25,26,27]. Our observations suggest that obesity or the consumption of a high-fat diet may alter endothelial responses to vascular stimuli in a way that mitigates endothelial dysfunction in this mouse model of preeclampsia, potentially through compensatory mechanisms that require further investigation.

The results on circulating vasoconstrictor levels add a layer of complexity to the obesity–preeclampsia paradigm. Circulating ET-1 levels are elevated in preeclampsia [41,42,43] and obesity [44,45], and we have previously shown that L-NAME administration significantly increases circulating ET-1 in pregnant mice [31]. However, in this study, obesity with superimposed L-NAME-induced preeclampsia resulted in a significant reduction in ET-1 compared to lean mice with L-NAME-induced preeclampsia. This decrease in ET-1 may explain the reduction in blood pressure and improved vasorelaxation also observed in these mice. This could indicate a potential protective effect of obesity in this specific context, although the lack of differences in sFLT-1 levels and CRP between the two groups suggests that other inflammatory and angiogenic pathways may remain unaffected in this model. Interestingly, sFLT-1 is significantly increased in preeclampsia [46,47,48,49] and appears to be positively correlated with BMI in pregnancy [50]. Adipose tissue has been shown to secrete sFLT-1, both in vivo in rats [51] and in vitro in cultured human adipose tissue biopsies [52]. Given this, it is not unreasonable to expect to see increased sFLT-1 in the obese mice due to their greater adiposity, but that was not observed here.

Placenta from the obese mice with superimposed preeclampsia were also significantly larger than those of lean mice with superimposed preeclampsia. This is consistent with human data showing a positive correlation between maternal BMI and placental weight [53]. Interestingly, we have previously shown that compared to normal pregnant mice, L-NAME significantly decreases placental weight [31]. This suggests that the increased placental weights observed here with obesity may not be a placental overgrowth but rather a rescue or compensatory aspect of obesity in the context of L-NAME. However, direct comparisons between normal pregnant and obese mice with superimposed preeclampsia are necessary to elucidate this further. Histological analysis of the placenta may provide additional insight. In the kidney, despite decreased *Hsd11b2*, *Nox4*, and *Fn1* expression with obesity, histological analysis revealed extensive glomerular damage, including swelling, increased cellularity, and narrowed Bowman’s space, suggesting significant renal pathology. The reduced expression of these markers may reflect a maladaptive response rather than protection, potentially indicating altered glucocorticoid signalling, oxidative stress regulation, and extracellular matrix remodelling that contribute to renal dysfunction in obese preeclamptic mice.

## 5. Conclusions

Our study provides novel data that challenge the assumption that obesity alone exacerbates the severity of symptoms of preeclampsia, suggesting instead that obesity may have complex, context-dependent effects. By employing more refined animal models, potentially with longer exposure to a high-fat diet or a greater increase in adiposity, we can continue to develop an understanding of the pathophysiology governing the interaction between obesity and preeclampsia.

## Data Availability

Data available upon reasonable request.

## Supplementary Materials

The following supporting information can be downloaded at https://www.mdpi.com/article/10.3390/nu17091586/s1, Figure S1. Effect of high fat diet feeding on dam weight gain; Figure S2. Systolic and diastolic blood pressure of lean and obese mice at D14.5 (A, B) and D17.5 (C, D); Figure S3. Vascular reactivity parameters for response of mesenteric arteries collected from obese and lean mice administered L-NAME; Figure S4. Effect of diet on fetal and placental size and weight split by fetal sex.
