# Supplementary material for: Investigating the Impact of Maternal Obesity on Disease Severity in a Mouse Model of Preeclampsia
Source: Nutrients. 2025 May 5;17(9):1586. doi: 10.3390/nu17091586 (PMC12073173; doi:10.3390/nu17091586)
Supplement: Supplementary file 1 [file nutrients-17-01586-s001.zip › nutrients-3591093-supplementary.pdf]

Supplementary figures

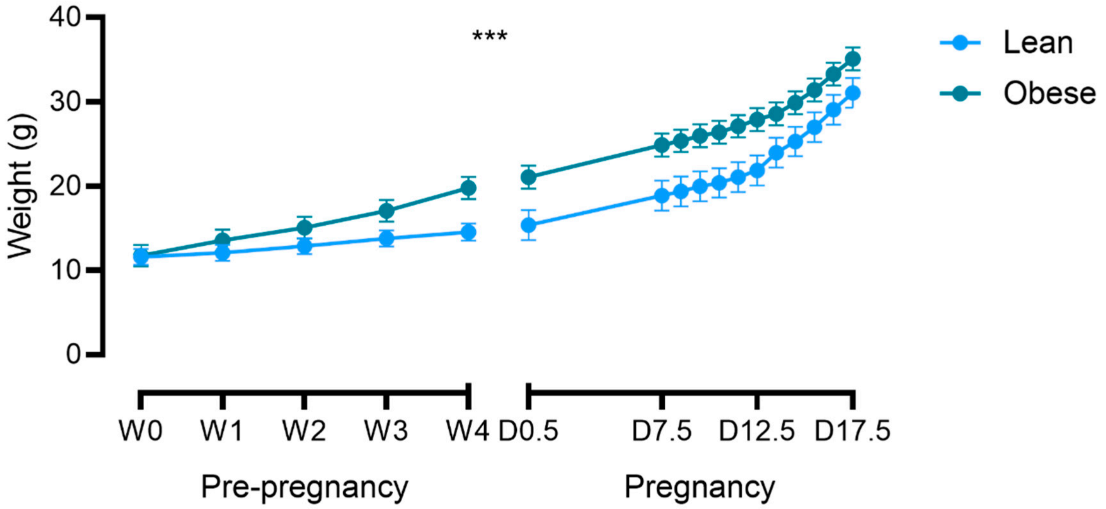

Figure S1. Effect of high fat diet feeding on dam weight gain. Mice were weighed weekly from week (W)0 to W4 pre-pregnancy and then on gestational day (D)0.5 and daily from D7.5-D17.5 of pregnancy. Mean ± SEM, n=10/group. ***p<0.001 assessed via two-way ANOVA with repeated measures

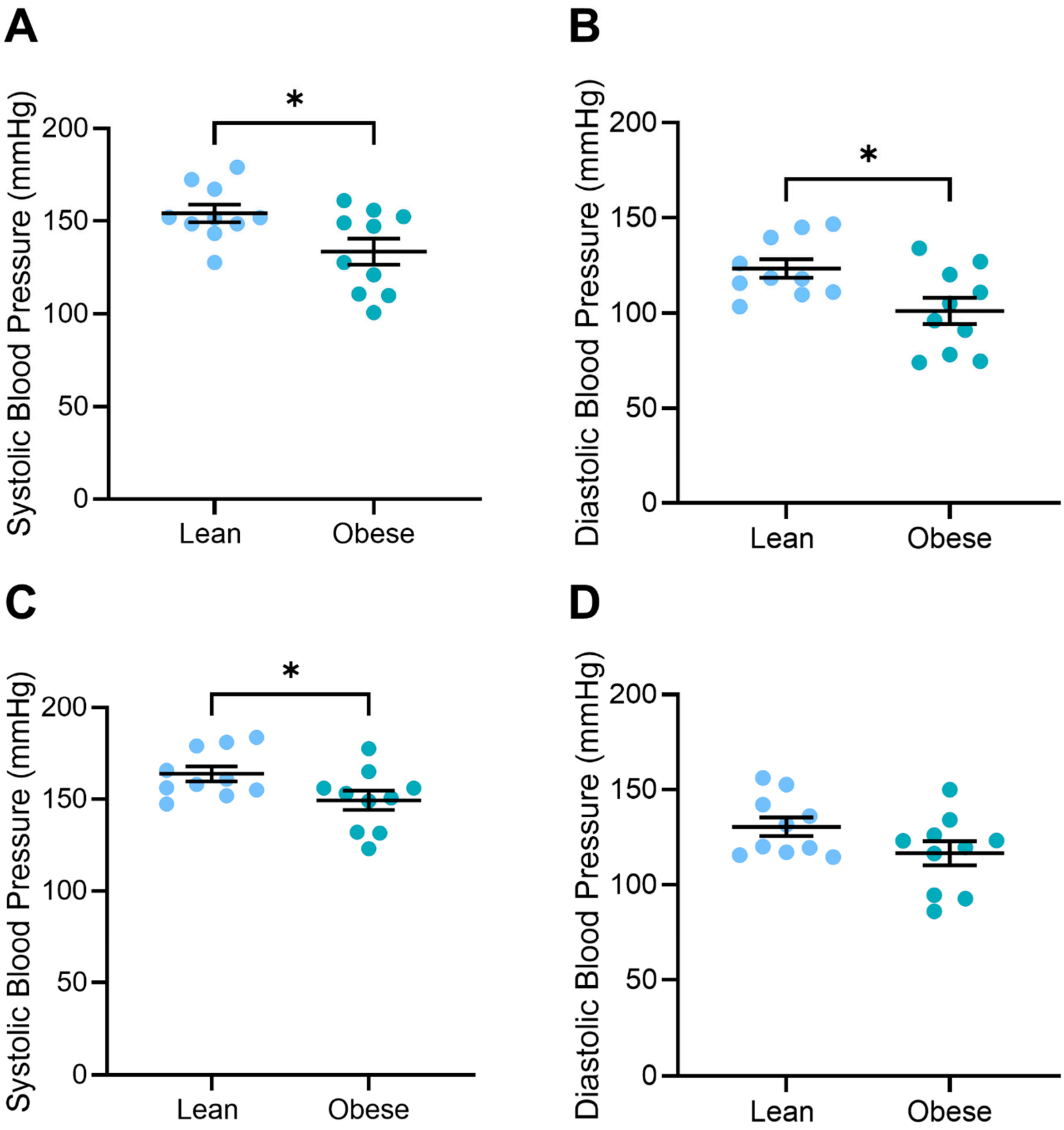

Figure S2. Systolic and diastolic blood pressure of lean and obese mice at D14.5 (A, B) and D17.5 (C, D). Blood pressure was measured via tail cuff plethysmography. At D14.5, systolic (A) and diastolic (B) blood pressure was lower in the obese dams compared to the lean mice. At D17.5, obese dams had significantly lower systolic blood pressure (C) but no difference in diastolic blood pressure (D) compared to the lean dams. Mean ± SEM, n=10/group. *p<0.05

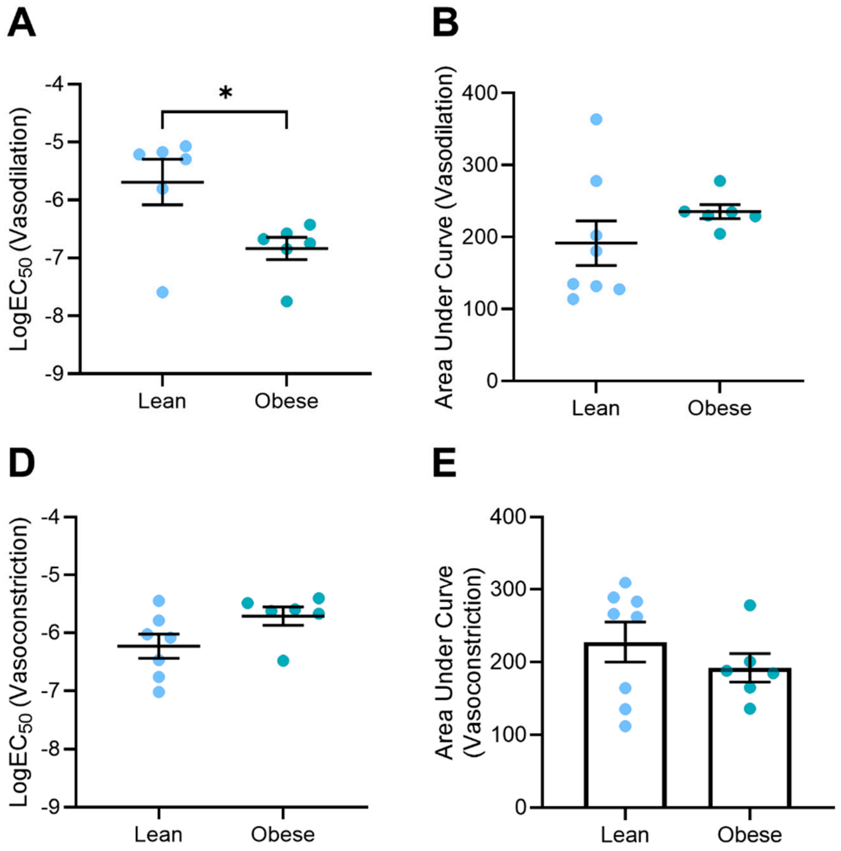

Figure S3. Vascular reactivity parameters for response of mesenteric arteries collected from obese and lean mice administered L-NAME. Corresponding graphs to the myograph dose-response curves presented in Figure 4: vasorelaxation to acetylcholine (A-C) and vasoconstriction to phenylephrine (D-F). Vasorelaxation LogEC50 was decreased in mesenteric arteries from obese dams compared to lean (A), but area under the curve (B) and maximum relaxation (Rmax; C) were not altered. Neither LogEC50 (D), area under the curve (E) nor maximum constriction (Emax) were altered in response to phenylephrine between the two groups. Mean ± SEM, n=6-7/group. *$p<0.05$

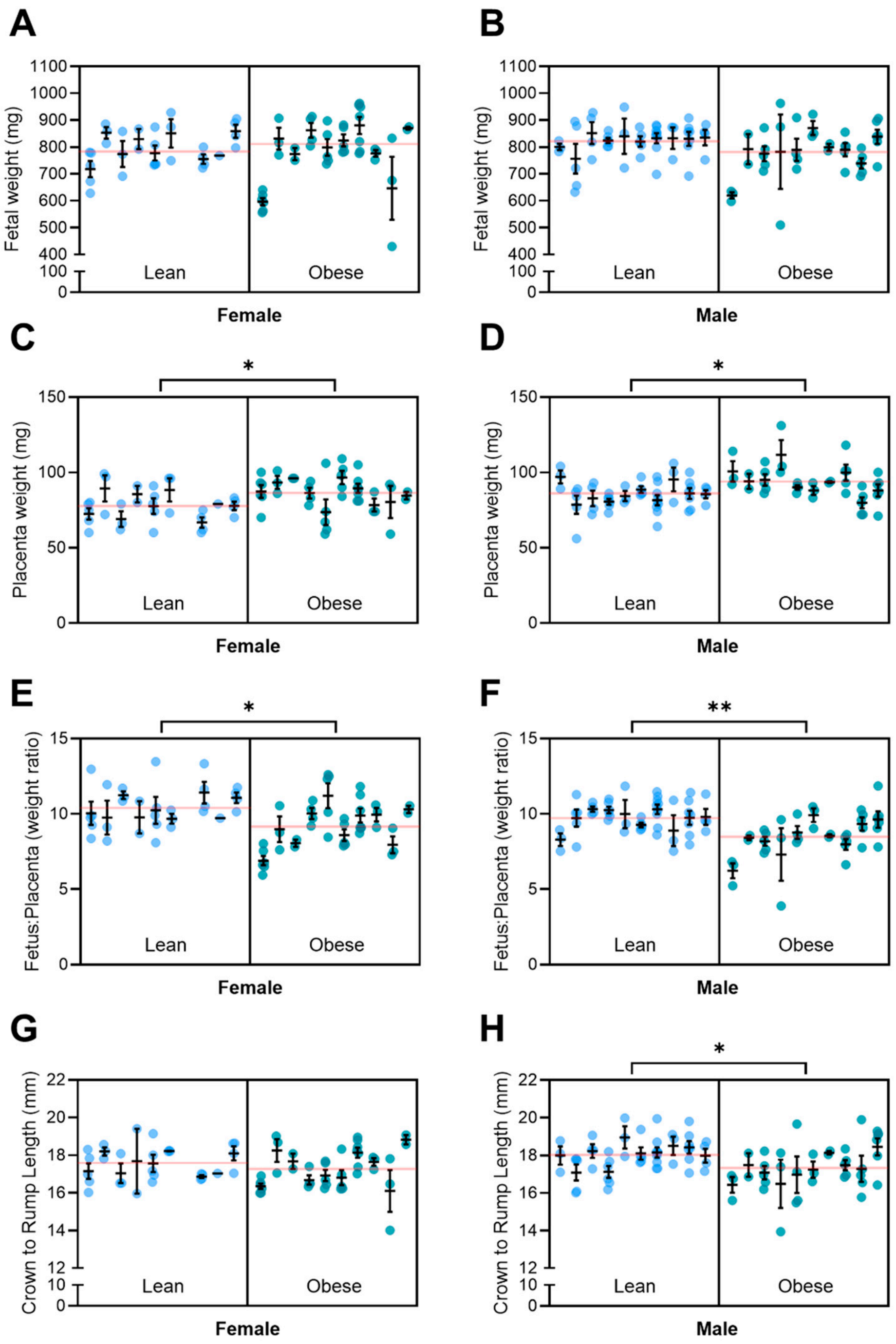

Figure S4. Effect of diet on fetal and placental size and weight split by fetal sex. Fetal weight was not significantly different between the litters of obese compared to lean dams for either female (A) or male (B) fetuses. Placental weight was significantly elevated (C, D), and fetal to placental weight ratio (E, F) decreased in the pups of both sex from the obese versus lean dams. Crown to rump length of female fetuses was not different between the groups, but male pups from obese dams had a lower crown to rump length compared to those from lean dams. Each sub column presents pups from a single dam. Red line across each box presents the mean of the group. Mean ± SEM, n=10 dams/group. *$p<0.05$, **$p<0.01$.
